# CANDLES, an assay for monitoring GPCR induced cAMP generation in cell cultures

**DOI:** 10.1186/s12964-014-0070-x

**Published:** 2014-11-04

**Authors:** Ashutosh Trehan, Emmi Rotgers, Eleanor T Coffey, Ilpo Huhtaniemi, Adolfo Rivero-Müller

**Affiliations:** Department of Physiology, Institute of Biomedicine, University of Turku, Turku, Finland; Department of Surgery and Cancer, Institute of Reproductive and Developmental Biology, Hammersmith Campus, Imperial College London, London, UK; Faculty of Natural Sciences and Technology, Åbo Akademi University, Turku, Finland; Turku Centre for Biotechnology, University of Turku and Åbo Akademi University, BioCity, Turku, Finland; Department of Biochemistry and Molecular Biology, Medical University of Lublin, Lublin, Poland

**Keywords:** cAMP, Bioassay, GPCR, Biosensor, Cell-cell interaction, Kinetics, CANDLES, Gap junctions, Connexin, FRET

## Abstract

**Background:**

G protein-coupled receptors (GPCRs) represent a physiologically and pharmacologically important family of receptors that upon coupling to Gα_S_ stimulate cAMP production catalyzed by adenylyl cyclase. Thus, developing assays to monitor cAMP production is crucial to screen for ligands in studies of GPCR signaling. Primary cell cultures represent a more robust model than cell lines to study GPCR signaling since they physiologically resemble the parent tissue. Current cAMP assays have two fundamental limitations: 1) absence of cAMP kinetics as competition-based assays require cell lysis and measure only a single time-point, and 2) high variation with separate samples needed to measure consecutive time points. The utility of real-time cAMP biosensors is also limited in primary cell cultures due to their poor transfection efficiency, variable expression levels and inability to select stable clones. We therefore, decided to develop an assay that can measure cAMP not only at a single time-point but the entire cAMP kinetics after GPCR activation in untransfected primary cells.

**Results:**

CANDLES (*C*yclic *A*MP i*N*direct *D*etection by *L*ight *E*mission from *S*ensor cells) assay for monitoring cAMP kinetics in cell cultures, particularly in primary cultures was developed. The assay requires co-culturing of primary cells with sensor cells that stably express a luminescent cAMP sensor. Upon GPCR activation in primary cells, cAMP is transferred to sensor cells via gap junction channels, thereby evoking a luminescent read-out. GPCR activation using primary cultures of rat cortical neurons and mouse granulosa cells was measured. Kinetic responses of different agonists to adrenergic receptors were also compared using rat cortical neurons. The assay optimization was done by varying sensor-test cell ratio, using phosphodiesterase inhibitors and testing cell-cell contact requirement.

**Conclusions:**

Here we present CANDLES assay based on co-culturing test cells with cAMP-detecting sensor cells. This co-culture setup allows kinetic measurements, eliminates primary cell transfections and reduces variability. A variety of cell types (rat cortical neurons, mouse granulosa cells and established cell lines) and receptors (adrenergic, follicle stimulating hormone and luteinizing hormone/chorionic gonadotropin receptors) were tested for use with CANDLES. The assay is best applied while comparing cAMP generation curves upon different drug treatments to untransfected primary cells.

**Electronic supplementary material:**

The online version of this article (doi:10.1186/s12964-014-0070-x) contains supplementary material, which is available to authorized users.

## Background

G protein-coupled receptors (GPCRs) constitute the largest family of cell surface receptors (>800 in humans), which regulate a plethora of functions in multicellular organisms owing to their diverse ligands that range from odors, photons, ions, pheromones, amino acids, peptides and neurotransmitters to hormones [1,2]. This inherent diversity in ligand subtypes also leads to activation of multiple cellular pathways both via G protein-dependent (Gα_S_, Gα_i/o_, Gα_q/11_, Gα_12/13_ and G_βγ_) and independent pathways [2–6]. One of the major pathways upon GPCR activation acts via coupling to Gα_S_ protein thereby activating adenylyl cyclase [7] that leads to conversion of adenosine triphosphate (ATP) to the secondary messenger, 3′,5′-cyclic adenosine monophosphate (cAMP) [8–10]. Thus the detection of cAMP constitutes an important readout for monitoring GPCR activation as well as for screening for potential ligands to GPCRs.

**Figure 1:**
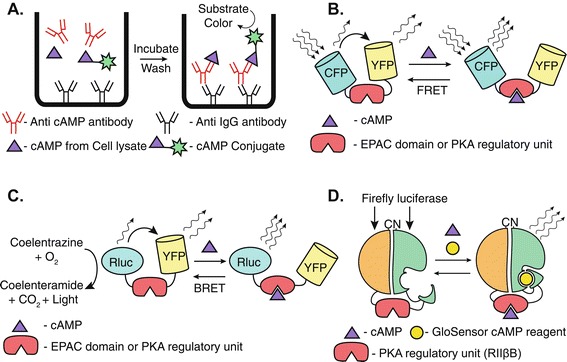
**Principles of commonly used cAMP assays. (A)** A colorimetric competitive enzyme-linked immunosorbent assay (ELISA) involving incubation of anti-IgG antibody-coated plates with peroxidase labeled cAMP (cAMP-conjugate), anti-cAMP antibody, and cell lysate containing endogenous cAMP. Endogenous cAMP produced upon GPCR stimulation in cells competes with cAMP conjugate for anti-cAMP antibody binding sites. After incubation and washing steps, cAMP-conjugate remaining in the well gives a colorimetric readout upon peroxidase substrate addition. **(B)** FRET-based sensor design containing cAMP binding domain from either PKA or EPAC protein fused between cyan fluorescent protein (CFP) and yellow fluorescent protein (YFP). In the absence of cAMP, there is FRET between CFP and YFP, whereas cAMP binding to the binding domain leads a conformational change resulting in loss of FRET between CFP and YFP. **(C)** BRET-based sensor design containing cAMP binding domain from either EPAC or PKA protein fused between *Renilla* luciferase (Rluc) and YFP. In the absence of cAMP, Rluc utilizes coelenterazine substrate to generate light, part of which is transferred via resonance (BRET) to YFP. Binding of cAMP to the sensor causes a conformational change, thereby abolishing BRET between Rluc and YFP. **(D)** Design of GloSensor-22F cAMP sensor (adapted from [31]). cAMP-binding domain from PKA regulatory subunit (RIIβB) is fused between *N*- and *C*-termini of a permuted firefly luciferase. Binding of cAMP to RIIβB favors a conformational change in two domains of luciferase, which in the presence of its substrate (GloSensor cAMP reagent) gives a luminescent read-out.

Primary cell cultures represent a biologically robust model over immortalized or transformed cell lines to study GPCR signaling, since the former are physiologically closer to their parent tissue with respect to their genetic integrity, receptor numbers, life span as well as metabolic pathways and regulatory control [11–13]. Additionally, primary cell cultures are more appropriate to study the function of a GPCR that is endogenously expressed in the cells at physiological levels, rather than the commonly employed method of exogenous receptor over-expression in cell lines. One of the major limitations of the frequently used assays for cAMP detection, especially in primary cell cultures, is the inability to kinetically monitor cAMP production, as majority of the methods are competition assays that require cell lysis, thus allowing only a single time-point measurement (see Figure 1A for a representative colorimetric competition assay) [14–18]. Moreover, to study the magnitude of cAMP production at different time points after GPCR activation, a different set of samples is needed, which further adds to the variability among separate time points, as has traditionally been done using immunoassays [17,18].

Recently, the focus in designing cAMP assays has shifted to the development of biosensor systems that can detect cAMP generation in real time in living cells [19]. These sensor proteins usually contain a cAMP-binding domain based either on Protein Kinase A (PKA) [20] or Exchange Protein Activated by cAMP (EPAC) [21] fused between two fluorescence resonance energy transfer (FRET) [22–28] or bioluminescence resonance energy transfer (BRET) [29,30] pairs. Binding of cAMP to PKA or EPAC domain causes a change in FRET or BRET ratio that evokes a live readout of GPCR activity (Figure 1B and 1C). Another useful system is the sensor (GloSensor-22F) that consists of a cAMP-binding domain of PKA (RIIβB) fused between the *N*- and *C*-termini of *Photinus pyralis* luciferase. Upon cAMP binding to the PKA domain, a conformational change allows the two domains of luciferase to attain a functional conformation and thus to metabolize luciferin (GloSensor cAMP reagent), giving a luminescent read-out (Figure 1D) [31]. However, the application of these methods to primary cell cultures is limited due to: (1) difficulties associated with transfecting primary cells, (2) the heterogeneous populations resulting from the variable expression of these sensor systems, and (3) the inability for selecting stable clones. The best solution to transfect these sensors in primary cells is to use viral transfection methods [32] (adeno-, lenti- or retroviruses) that require at least biosafety level 2 (BSL-2) facilities and the need of species-specific viruses (e.g. adenoviruses), yet points 2 and 3 still apply.

To overcome the aforementioned problems, we introduce a new method for monitoring cAMP generation, especially from primary cell cultures. Our method involves generation of a separate stable sensor cell line that expresses a cAMP sensor (GloSensor 22F) in co-culture with the cells under study (expressing the GPCR whose function is to be studied), thereby eliminating the need to either transfect primary cells or to use a different set of samples for different time points. GPCR stimulation in the cells under study leads to cAMP generation, which is then transferred to the co-cultured sensor cells. The detection of cAMP by the sensor cells causes a change in the conformation of the cAMP sensor protein, which in the presence of a luciferin substrate gives a luminescent readout of GPCR activation-dependent activity (Figure 1D). Since the assay involves indirect detection of cAMP produced by the primary cells as a luminescent readout by the co-cultured sensor cells, we named the assay as the CANDLES (*C*yclic *A*MP i*N*direct *D*etection by *L*ight *E*mission from *S*ensor cells) assay.

## Results

### Proof of concept

**Figure 2:**
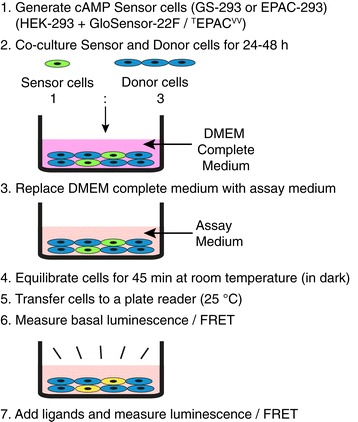
**CANDLES Assay protocol.** The CANDLES assay involves co-culture of sensor cells (GS-293 or EPAC-293) with donor cells (primary cells or cell lines). DMEM complete medium is replaced with freshly prepared assay medium and cells are pre-incubated (in dark for GS-293 cells) before luminescence/ FRET measurements can be read upon ligand stimulation.

Previous studies have shown that gap junction channels [33–35], which serve as intercellular communication channels between neighboring cells, allow transfer of small molecules and metabolites (<1kDa) including cAMP [36–40]. We thought of utilizing this property and hypothesized that cAMP produced upon stimulation of GPCRs in donor cells (primary cells or established cell lines) could be detected by a separate sensor cell line (cAMP sensor cells) if they are co-cultured (Figure 2).

Prior to isolating primary cells from animals, we first tested two established cell lines with rarely expressed GPCRs in donor cells: KK-1 cells [41] that endogenously express luteinizing hormone/chorionic gonadotropin receptor (LHCGR) and human embryonic kidney-293 (HEK-293) cells stably-transfected with human follicle-stimulating hormone receptor (FSHR) [from here forward referred to as FSHR-293]. The underlying concept behind testing FSHR and LHCGR was that the donor cells express these receptors while the sensor cells do not.

**Figure 3:**
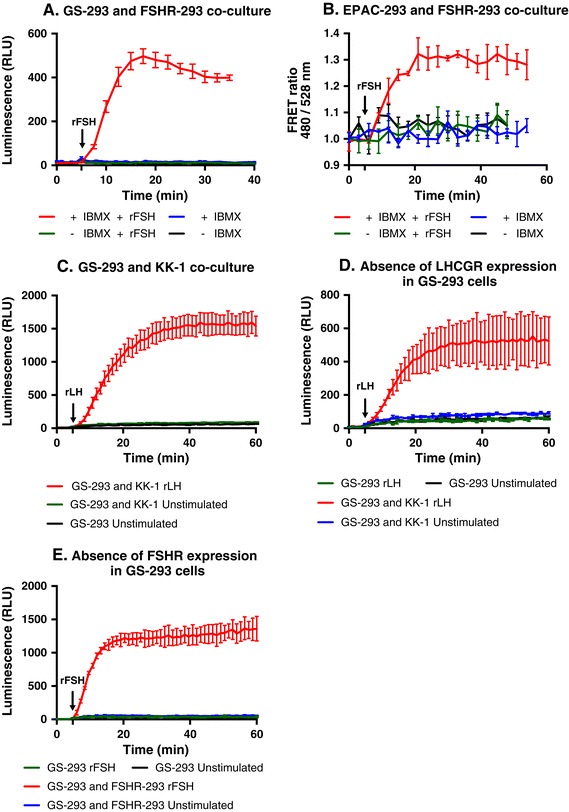
**Detection of cAMP generated by donor cells using sensor cells. (A)** Preincubation of co-cultures of GS-293 (50,000 cells) with FSHR-293 (50,000 cells) for 45 minutes with assay medium either in the presence or absence of IBMX and monitoring luminescence after addition of rFSH (200 mIU/ml). GS-293 cells could detect cAMP production upon stimulation of FSHR-293 cells with rFSH only in the presence of IBMX. In the absence of IBMX, no luminescent signal upon FSHR-293 stimulation could be detected. Data represented as mean of triplicates for one representative experiment ± standard error of the mean (SEM). **(B)** EPAC-293 and FSHR-293 co-cultures (50,000 cells each) were stimulated with rFSH (200 mIU/ml) in the presence or absence of IBMX. A change in FRET ratio (480/528 nm) was observed after rFSH stimulation. EPAC-293 cells could detect cAMP production from co-cultured FSHR-293 cells only in the presence of IBMX. Data represented as mean of triplicates for one representative experiment (± SEM). **(C)** Co-culture of GS-293 (25,000 cells) with KK-1 (75,000 cells) and monitoring luminescence after addition of rLH (100 ng/ml). Data represented as mean of triplicates for one representative experiment (± SEM). **(D and E)** Absence of LHCGR and FSHR expression in GS-293 cells is shown by luminescence values upon rLH (100 ng/ml) and rFSH (200 mIU/ml) stimulation being similar to unstimulated GS-293 control. Stimulated co-culture of GS-293 cells with either KK-1 or FSHR-293 cells was used as positive control for LHCGR and FSHR expression, respectively. Unstimulated co-cultures of GS-293 with either KK-1 or FSHR-293 were used as negative control. Data represented as mean of duplicates for one representative experiment [± standard deviation (SD)]. All the experiments have been independently repeated at least three times. (Relative Light Unit: RLU).

We compared two different sensors, a luminescence based sensor (GloSensor-22F) and a FRET based cAMP sensor (^T^EPAC^VV^) by stably transfecting these sensors in HEK-293 cells, thereby generating two sensor cell lines, GS-293 and EPAC-293, respectively (see Methods). HEK-293 cells were chosen to generate sensor cells since they express moderate levels of gap junctions [35,42]. We tested if GS-293 or EPAC-293 cells could sense the cAMP generated by FSHR-293 cells stimulated by follicle-stimulating hormone (FSH). Since the cAMP generated by donor cells is degraded by endogenous phosphodiesterases, we used a non-specific phosphodiesterase inhibitor, 3-isobutyl-1-methylxanthine (IBMX), to prevent cAMP degradation. In the absence of IBMX, GS-293 cells (Figure 3A) or EPAC-293 cells (Figure 3B) cannot detect cAMP generated upon stimulation of FSHR-293 cells with recombinant human (r) FSH (Merck-Serono). Unstimulated co-cultures of sensor and donor cells either in presence or absence of IBMX were used as controls to observe the effect of IBMX alone. It is therefore crucial for this assay to use phosphodiesterase inhibitor so that cAMP generated by donor cells will not be degraded before the sensor cells can detect it. We then tested different doses of IBMX, where 100 μM generated a better signal over background ratio (Additional file 1: Figure S1) and thus was used for all further experiments.

As expected, both sensor cell lines were able to detect cAMP generated from co-cultured FSHR-293 cells upon stimulation with rFSH (Figure 3A and 3B). However, we chose the luminescent sensor cells (GS-293) over the FRET-based sensor cells (EPAC-293) for further experiments since we could not detect a dose-dependent change in FRET ratio with the EPAC-293 sensor cells when the co-cultured FSHR-293 cells were stimulated with increasing doses of rFSH (Additional file 2: Figure S2). Moreover, luminescence measurements were not associated with photobleaching of the sensor and thereby did not require additional normalization controls.

We then tested a cell line, KK-1 cells, which expresses an endogenous GPCR, in this case the LHCGR. As shown in Figure 3C upon stimulation with recombinant luteinizing hormone (rLH), the GS-293 cells were able to detect the cAMP generation of neighboring KK-1 cells. Unstimulated co-culture of GS-293 and KK-1 cells as well as GS-293 cells cultured alone were used as controls. GS-293 cells did not respond to either ligand when cultured alone (Figure 3D and 3E).

### Optimizing the ratio of sensor cells to donor cells

**Figure 4:**
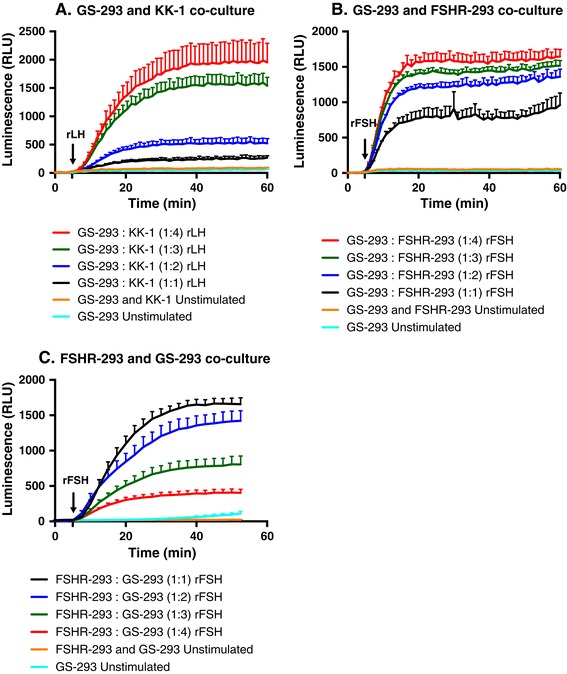
**Optimization of the ratio of sensor (GS-293) to donor cells for maximal luminescence (cAMP generation). (A)** Cell seeding density of GS-293 cells: KK-1 cells in increasing ratio from 1:1 to 1:4 (25,000 cells of each for 1:1 ratio). **(B)** Cell seeding density of GS-293: FSHR-293 cells in increasing ratio from 1:1 to 1:4 (25,000 cells of each for 1:1 ratio). **(A and B)** An increased luminescent signal [proportional to cAMP production upon rLH (100 ng/ml) or rFSH (200 mIU/ml) stimulation] was detected with increasing number of donor cells (KK-1 or FSHR-293). **(C)** Cell seeding density of FSHR-293: GS-293 cells in increasing ratios from 1:1 to 1:4 (25,000 cells of each for 1:1 ratio) and a decrease in luminescent signal was observed for an increasing number of GS-293 cells. **(A, B and C)** Data represented as mean of triplicates for one representative experiment (± SEM; positive direction) with at least three independent repeats.

To find the optimal conditions for detecting high cAMP signal, we first kept the number of sensor cells (GS-293) constant and increased the number of donor cells (KK-1 or FSHR-293) in increasing ratios (sensor cells: donor cells in increasing ratio from 1:1 to 1:4). As expected, cAMP signal detected by GS-293 cells increased with increasing ratio of donor cells (Figures 4A and 4B). Moreover, beyond a ratio of 1:3 of sensor to donor cells, the proportionate increase in luminescent signal was less marked, and hence the optimal starting sensor to donor cell ratio for using this method for other cell types and receptors was considered to be around 1:3.

However, when we increased the number of sensor cells (GS-293) and kept the number of donor cells (FSHR-293) constant, we observed that cAMP detection via GS-293 cells decreased as the number of GS-293 cells increased (Figure 4C) upon stimulation of cells with rFSH (200 mIU/ml). This is likely due to decreasing number of cell-cell contacts between donor and sensor cells as an increasing number of sensor cells facilitate more cell-cell contacts among themselves than between sensor and donor cells. Hence increasing the number of sensor cells may not increase the luminescent read-out. Unstimulated co-culture of GS-293 with either KK-1 cells or FSHR-293 cells as well as GS-293 cells cultured alone were used as controls for all the aforementioned experiments showing no cAMP responses.

### Cell-to-cell contact is needed for cAMP detection

**Figure 5:**
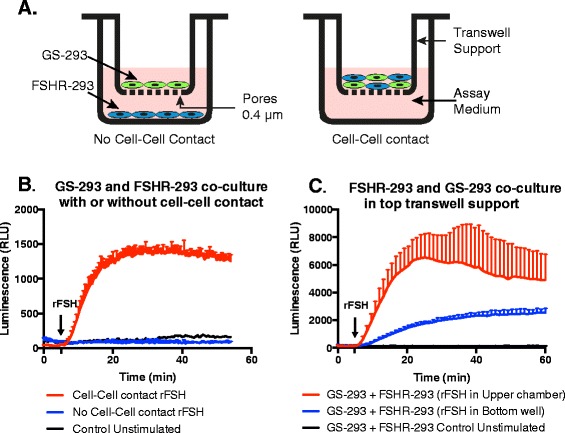
**GS-293 cells require cell-cell contact to detect cAMP from donor cells. (A)** GS-293 cells were grown on transwell permeable supports and FSHR-293 cells were grown on the bottom of a separate well. Prior to experiment, the transwell chamber with the GS-293 cells was placed above the FSHR-293 cells and medium was replaced with assay medium (referred to as ′No cell-cell contact” configuration). GS-293 and FSHR-293 cells were also co-cultured in the transwell permeable support wells (referred to as “Cell-Cell contact” configuration). **(B)** In “No cell-cell contact” configuration, GS-293 cells were unable to detect cAMP production from FSHR-293 cells stimulated with rFSH (200 mIU/ml) and the luminescent signal detected was comparable to unstimulated GS-293 cells (control). The cell-cell contact configuration however yielded much higher luminescence, proving the detection of cAMP by GS-293 cells from donor cells requires cell-cell contact. **(C)** In cell-cell contact configuration, rFSH stimulation in the upper chamber yielded a higher response than rFSH stimulation in the bottom well due to higher effective concentration of rFSH in the former. **(B and C)** Data represented as mean of duplicates for one representative experiment (± SD; positive direction) with at least three independent repeats.

We had so far co-cultured sensor and donor cells in the same well such that both cell types have cell-cell contacts. To further test whether a decreasing number of cell-cell contacts between sensor and donor cells was responsible for a decrease in signal as shown in Figure 4C, we tested the effect of complete abolition of contacts between the sensor and donor cells. To this end, we cultured FSHR-293 and GS-293 cells in isolation using 24-well permeable support transwells (containing pores of 0.4 μm for diffusion of molecules) in DMEM complete medium (Figure 5A). After a one-day culture, DMEM complete medium was discarded. 300 μl of assay medium was added to FSHR-293 cells, and GS-293 cells growing in transwell permeable support were placed on top of the FSHR-293 wells. Finally 100 μl of assay medium was also added on top of transwell permeable supports. The porous membrane ensures that the cells can still interact in a paracrine fashion through the medium rinsing both cell types. The cells were then stimulated with rFSH (200 mIU/ml) and compared with stimulation of GS-293 cells and FSHR-293 cells with rFSH that were co-cultured with cell-cell contacts. The data in Figure 5B shows that cell-cell contact is necessary for detection of cAMP by GS-293 cells. Separation of GS-293 and FSHR-293 cells with transwell permeable support leads to loss of cAMP detection by GS-293 cells, comparable to unstimulated control (GS-293) cells.

To test whether the permeable membrane could allow the movement of small molecules, or even large glycoprotein hormones like rFSH, FSHR-293 and GS-293 were co-cultured in transwell chamber (15,000 cells each) for 48 hours. The cells were similarly tested in assay medium and rFSH was added in either the top chamber or the bottom well. Figure 5C shows that cAMP signal from FSHR-293 cells could be detected by GS-293 cells, the only difference being stimulation in top chamber (containing 100 μl of assay medium) was more pronounced than in the bottom well (containing 300 μl of assay medium) due to differences in effective concentration of the hormone in the two chambers. Unstimulated co-culture of FSHR-293 with GS-293 cells was used as negative control.

### Gap junction channels mediate cAMP transfer from donor to sensor cells

**Figure 6:**
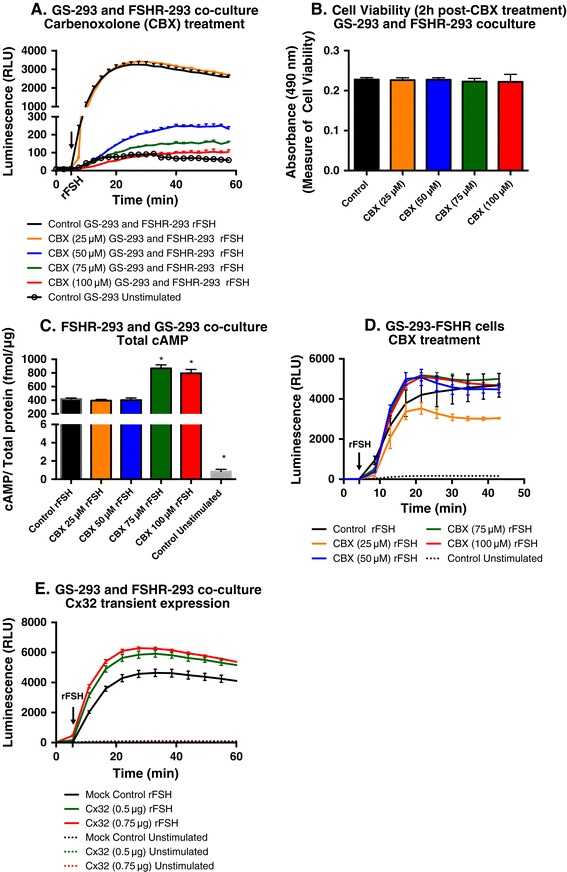
**Gap junctions mediate transfer of cAMP from donor to sensor cells. (A)** CBX (25, 50, 75 and 100 μM) preincubated co-cultures of GS-293 with FSHR-293 (50,000 cells each) were stimulated with rFSH (200 mIU/ml). Increasing doses of CBX lead to decreasing luminescence, with 100 μM CBX reducing the luminescence to levels similar to unstimulated GS-293 control cells. Data represented as mean of triplicates for one representative experiment ± SEM (positive direction only) with at least three independent repeats. **(B)** GS-293 and FSHR-293 co-cultures (50,000 cells each) were incubated with CBX (25, 50, 75 and 100 μM) for 2h in assay medium while the control samples had only assay medium without CBX. Cell viability 2h post-CBX treatment shows no statistical difference among samples using one-way ANOVA (p-value =0.9891). **(C)** Total cAMP concentration in co-cultures of GS-293 and FSHR-293 (150,000 cells each) as assessed by ELISA following stimulation with rFSH (200 mIU/ml) for 20 min. Total cAMP concentration in cells treated with 75 μM and 100 μM CBX was higher (p < 0.0001) than stimulated control while those with 25 μM and 50 μM CBX are similar to the stimulated control. **(D)** rFSH (200 mIU/ml) stimulation of GS-293-FSHR (50,000 cells) preincubated with higher doses of CBX (50, 75 and 100 μM) moderately increased cAMP production as compared to GS-293-FSHR control cells (preincubated with assay medium without CBX). **(E)** Co-cultures of GS-293 and FSHR-293 were transfected with increasing amounts of Cx32 plasmid. Co-culture of FSHR-293 and GS-293 transfected with pcDNA3.1 mock plasmid was used as control. Cx32 expression lead to a statistically significant (p-value =0.0017) increase in detection of cAMP by the sensor cells as compared to control stimulated cells. **(B, C, D and E)** Data represented as mean of triplicates for one representative experiment (± SEM) with at least three independent repeats.

To further explore the molecular components of cell-to-cell contact that were responsible for transfer of cAMP from donor to sensor cells, we tested the role of gap junctions. Carbenoxolone (CBX), a gap junction inhibitor [43,44], was added to co-cultures of GS-293 and FSHR-293 cells (Figure 6A). GS-293 and FSHR-293 co-cultures were preincubated for 1h with different doses of CBX in assay medium, followed by stimulation with rFSH (200 mIU/ml). Figure 6A shows a dose–dependent decrease in luminescence from the sensor cells, while the lowest dose (25 μM) resulted in similar values to positive control (GS-293 and FSHR-293 co-culture without CBX and stimulated with rFSH), the highest CBX dose (100 μM) completely abolished cAMP detection as the signal levels were comparable to those of unstimulated control (GS-293) cells.

In order to eliminate that the effect was due to cytotoxicity upon CBX treatment, cell viability was assessed 2h post-CBX treatment. As shown in Figure 6B, CBX treatment did not alter cell viability and the dose-dependent decrease in luminescent signal as seen in Figure 6A cannot be attributed to cell death.

To further examine whether the drop in luminescence in Figure 6A was only due to inhibition of cAMP transfer and not due to decrease in cAMP production, we first assessed the absolute cAMP concentrations after stimulation of GS-293 and FSHR-293 co-cultures via a cAMP enzyme-linked immunosorbent assay (ELISA) kit. GS-293 and FSHR-293 cells were co-cultured in 6-well plates. Prior to ELISA, cells were incubated with different concentrations of CBX in assay medium (without GloSensor cAMP reagent) for 1h at room temp and subsequently stimulated with rFSH (200 mIU/ml) for 20 min. Cells were lysed and cAMP concentrations were determined according to manufacturer’s instructions. GS-293 and FSHR-293 co-cultures incubated in assay medium (without CBX and GloSensor cAMP reagent) were used as controls. Figure 6C shows that the total cAMP content in the co-cultures is either very similar (for 25 μM and 50 μM CBX) or higher (for 75 μM and 100 μM CBX) to the stimulated (rFSH) control. The observed increase in cAMP at higher CBX doses might be due to blockage of the physiological mechanism of cAMP transfer to the neighboring cells and leakage to the cell culture medium, thereby causing higher retention of cAMP in treated cells. This suggests that the drop in signal observed in Figure 6A was not due to a decrease in cAMP production but rather due to inhibition of cAMP transfer via gap junctions.

Since cAMP ELISA can determine only a terminal time point, we decided to use our sensor GS-293 cells stably transfected with human FSHR (hereby called GS-293-FSHR cells) to determine the effect of CBX on cAMP production in the same cells containing both the receptor and the sensor with kinetics mode. Figure 6D shows no significant changes in cAMP generation in the presence of CBX. As in Figure 6C, higher doses of CBX have a tendency to moderately increase cAMP production. This further supports that cAMP production is not reduced upon CBX treatment. All these observations point to the conclusion that it is indeed the blockage of cAMP transfer from donor to sensor cells upon inhibition of gap junction communication that caused a dose-dependent reduction of luminescent signal (Figure 6A).

To further confirm whether gap junction channels, which are composed of connexin proteins, were responsible for cAMP transfer from donor to senor cells, co-cultures of FSHR-293 and GS-293 cells were transiently transfected with increasing amounts of human connexin-32 (Cx32) plasmid. The total amount of transfected DNA was kept constant at 0.75 μg DNA/well by addition of pcDNA3.1 mock plasmid. The expression of Cx32 (fluorescently tagged with mEmerald) was verified by using EVOS fluorescent microscope (Additional file 3: Figure S3). Figure 6E shows that overexpressing Cx32 increases the cAMP detection by the sensor cells as compared with mock-transfected cells (control). The increase in cAMP transfer is statistically significant to control (p-value =0.0017) as calculated by comparing the area under curve of luminescence values for different samples using one-way analysis of variance (ANOVA). The area under curve (in arbitrary units ± SEM) for different samples was Cx32 (0.75 μg): 384000 ± 5400, Cx32 (0.5 μg): 358000 ± 13000 and Cx32 (mock control): 279000 ± 16000. Thus we conclude that the mechanism of transfer of cAMP from donor cells to sensor cells is mediated via gap junctions.

### Dose-dependent detection of cAMP from donor cells by GS-293 sensor cells

**Figure 7:**
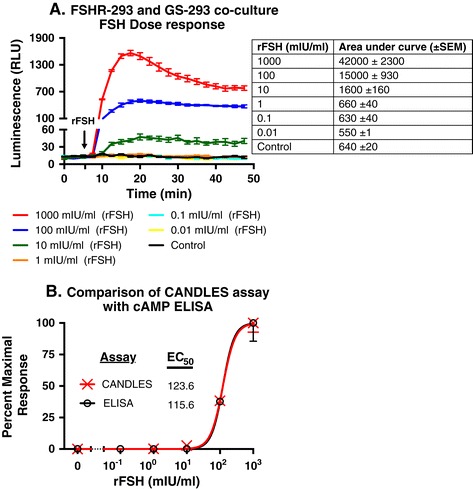
**Comparison of dose-response curves derived using CANDLES or cAMP ELISA. (A)** An increasing stimulation of FSHR-293 (50,000 cells) with rFSH (0.01 mIU/ml to 1000 mIU/ml) also yields a dose-dependent increase in luminescent signal from co-cultured GS-293 (50,000 cells). The adjoining table shows the area under curve of luminescence values for different rFSH doses. **(B)** Luminescence values (RLU) in Figure 7A (20 min after stimulation) were used to calculate EC_50_ for CANDLES (123.6 mIU/ml). A cAMP ELISA kit was used to compare EC_50_ values with CANDLES. FSHR-293 cells (150,000 cells) stimulated with similar doses of rFSH (0.1 mIU/ml to 1000 mIU/ml) for 20 min were used to calculate cAMP values using ELISA. Data were normalized to percentage of maximal responses and curves were fitted using four-parameter logistic curve. EC_50_ values calculated using CANDLES (123.6 mIU/ml) are very similar to those calculated using a cAMP ELISA kit (115.6 mIU/ml) and there is no statistical difference (p = 0.9885). **(A and B)** Data represented as mean of triplicates for one representative experiment (± SEM) with at least three independent repeats.

We also tested whether the amount of luminescent signal generated by GS-293 cells follows an expected dose-dependent increase when we stimulate the co-cultured donor cells (FSHR-293) with increasing amounts of rFSH. Figure 7A shows that the GS-293 response indeed is proportional to the increase in cAMP production from FSHR-293 cells following stimulation with different doses of rFSH. Unstimulated FSHR-293 and GS-293 co-culture was used as control. The adjoining table in Figure 7A also shows the area under curve (in arbitrary units) for the luminescence values detected at different rFSH concentrations. EC_50_ values were calculated from luminescence (Relative Light Unit: RLU) using a single time point, at 20 min after FSH stimulation, and compared with EC_50_ values calculated using a traditional cAMP ELISA kit of FSHR-293 cells grown in 6-well plates and stimulated with increasing doses of rFSH for 20 min. As shown in Figure 7B, EC_50_ values from CANDLES assay (123.6 mIU/ml) are comparable to those calculated using cAMP ELISA kit (115.6 mIU/ml).

### Detection of cAMP production from primary cell cultures

**Figure 8:**
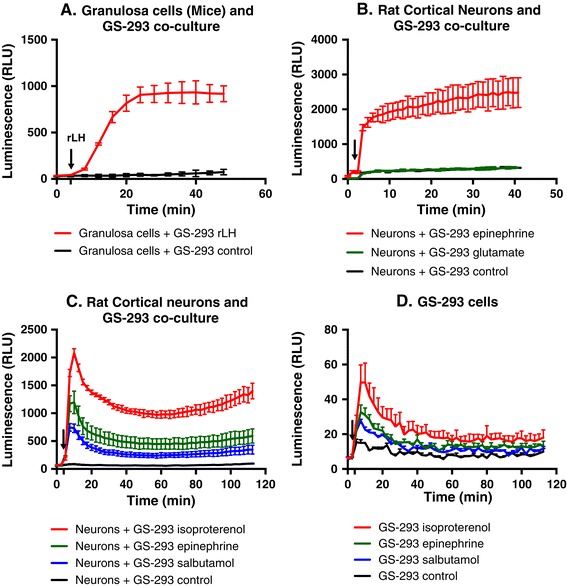
**Monitoring cAMP generation in primary cells with GS-293 cells. (A)** Mouse granulosa cells were co-cultured with GS-293 (100,000 cells). GS-293 detected cAMP generated by rLH (100 ng/ml) stimulation of LHCGR in granulosa cells. **(B)** Rat cortical neurons were co-cultured with GS-293 (100,000 cells) and stimulated with epinephrine or glutamate. Epinephrine led to generation of cAMP, while glutamate resulted in no response. **(A and B)** Data represented as mean of duplicates for one representative experiment (± SD) with at least three independent repeats. **(C)** Rat cortical neurons were co-cultured with GS-293 (100,000 cells) and stimulated with isoproterenol (100 nM), epinephrine (100 nM) and salbutamol (100 nM) to compare their different kinetic responses upon stimulation of adrenergic receptors. **(D)** GS-293 cells also express adrenergic receptors as can be seen in response to isoproterenol, epinephrine and salbutamol stimulation, although the response to stimulation is minimal in comparison to cortical neurons (compare the Y-axis with Figure 8 **C**). **(C and D)** Data represented as mean of triplicates for one representative experiment (± SEM) with at least three independent repeats.

GS-293 cells were co-cultured with two completely different primary cell types (mouse granulosa cells and rat cortical neurons) in order to test our method for a variety of cell-specific receptors. Granulosa cells endogenously express LHCGR and we selected rat cortical neurons as a blind test and therefore tested them for the expression of adrenergic receptors and glutamate receptors. Figure 8A depicts that stimulation of granulosa cells with rLH (100 ng/ml) resulted in stimulation of LHCGR and the downstream generation of cAMP, which in turn was detected by GS-293 cells. Similarly, the stimulation of rat cortical neurons (Figure 8B) by epinephrine showed the presence of adrenergic receptors whereas the stimulation by glutamate resulted in no detectable responses. Unstimulated co-cultures of GS-293 with either granulosa cells or neurons were used as negative controls. The above experiments confirm the utility of our method in kinetic measurements of cAMP production in primary cell cultures.

CANDLES assay was also used to compare the kinetics of different agonists for adrenergic receptors in rat cortical neurons (Figure 8C). Epinephrine was used as a non-selective agonist for α_1_, α_2_, β_1_ and β_2_ adrenergic receptor activation, and isoproterenol selectively activates β_1_ and β_2_ adrenergic receptors, while salbutamol activates only β_2_ adrenergic receptors. Although epinephrine, isoproterenol and salbutamol also stimulated GS-293 cells alone (Figure 8D), confirming the presence of endogenous adrenergic receptors in GS-293 cells, the signal (luminescence) was very low in comparison to what was observed for cortical neurons (compare Y-axes in Figures 8C and 8D). Hence, the GS-293 cells could still be used as sensor cells. Unstimulated co-culture of GS-293 with neurons and GS-293 cells cultured alone were used as negative controls.

### Adapting GS-293 cells from DMEM/F12 medium to other commonly used media

**Figure 9:**
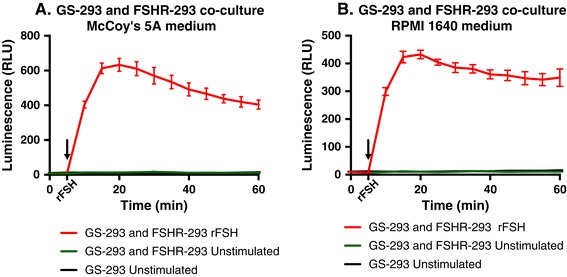
**Adapting GS-293 cells to grow in RPMI 1640 and McCoy’s 5A media.** Both GS-293 and FSHR-293 cells were step-wise adapted to grow in either McCoy’s 5A or RPMI 1640 media. After adapting cells, co-cultures of GS-293 (50,000 cells) and FSHR-293 (50,000 cells) were grown in either medium. **(A and B)** The CANDLES assay, performed after adapting the cells, can detect cAMP from co-cultured donor cells. Data represented as mean of triplicates for one representative experiment (± SEM) with at least three independent repeats.

GS-293 cells have so far been grown in DMEM/F12 medium. To ensure proper growth conditions of the sensor cells in co-cultures with different kind of cells growing in their own respective medium, we chose to adapt sensor cells in two of the most commonly used cell culture media; McCoy’s 5A and RPMI 1640. First, GS-293 cells were grown in medium containing 20% McCoy’s 5A and 80% DMEM/F12 medium for one week. In the successive weeks the concentration of McCoy’s 5A medium was increased to 40, 60, 80 and 100%. Similarly, GS-293 cells were also adapted in RPMI 1640 medium. Likewise, FSHR-293 cells were adapted in both McCoy’s 5A and RPMI 1640 media. CANDLES assay was then performed with co-cultures of GS-293 and FSHR-293 cells adapted in either 100% McCoy’s 5A medium or 100% RPMI 1640 medium. The assay medium was modified to contain a 1:1 ratio of CO_2_-independent medium with either McCoy’s 5A medium or RPMI 1640 medium. As shown in Figures 9A and 9B, sensor cells could be easily adapted to different media, thereby ensuring optimal growth conditions to that of the donor cell type of choice and hence can follow cAMP production upon stimulation of co-cultured donor cells. Unstimulated co-culture of GS-293 with FSHR-293 cells as well as GS-293 cells cultured alone were used as controls.

### Comparison of cAMP accumulation using CANDLES with real-time cAMP production

**Figure 10:**
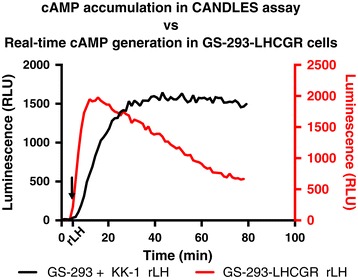
**Comparison of kinetics of cAMP generation using CANDLES with instantaneous cAMP production.** cAMP accumulation detected by GS-293 (25,000 cells) upon stimulation of co-cultured KK-1 (75,000 cells) with rLH (100 ng/ml) (black line, left Y-axis) using CANDLES. GS-293 cells stably transfected with a plasmid carrying the *Lhcgr* cDNA (GS-293-LHCGR) and stimulated with rLH (100 ng/ml) depicts real-time cAMP present in the cells (red line, right Y-axis).

The CANDLES assay measures cAMP accumulation over a period of time due to addition of non-specific phosphodiesterase inhibitor, IBMX. Therefore, the shape of the luminescence curve reveals cAMP accumulation over time rather than instantaneous cAMP production. We used GS-293 cells stably transfected with the LHCGR (GS-293-LHCGR) to obtain instantaneous cAMP kinetics after rLH (100 ng/ml) stimulation (Figure 10; right Y-axis, red line) in assay medium without IBMX. GS-293 cells co-cultured with KK-1 cells (natively expressing LHCGR) were used to obtain cAMP accumulation curve using CANDLES assay (Figure 10; left Y-axis, black line). As shown in Figure 10, cAMP accumulation saturates after apparent LHCGR desensitization.

## Discussion

The coupling of many GPCRs upon ligand activation to Gα_S_ leads to activation of adenylyl cyclase that catalyzes the production of cAMP [9,10]. Given the massive importance of GPCR signaling in pharmacology, many cAMP assays for screening ligands as well as to study the GPCR signaling have been designed. There are many model systems in which GPCR signaling can be studied, ranging from *in vivo* mouse models to *in vitro* cell culture systems using established cell lines (transformed or immortalized). Primary cell cultures using freshly isolated tissues from animal models or clinical samples represent a biologically relevant system to study GPCR signaling over immortalized or transformed cell lines, since the former retain most of their physiological functions and regulatory controls. However, the currently available methods for monitoring cAMP production, especially on primary cells, suffer from two major drawbacks. First, their inability to measure the kinetics of cAMP production since the majority of them are competition-based and hence require cell lysis after ligand stimulation to measure intracellular cAMP, thereby measuring only one single time-point. Second, it is difficult to transfect primary cells by most methods (except viral transfections) with new fluorescent or luminescent cAMP sensor encoding plasmids, which can ideally measure cAMP kinetics. Although viral transfections are highly efficient, they are labor-intensive, require special safety regulations and might only infect species-specific cells (e.g. adenoviruses), something that our assay does not require as mouse, rat and human cells were used in our studies.

Our CANDLES assay is able to kinetically monitor cAMP production in primary cell cultures upon specific GPCR stimulation by co-culturing them with the cAMP-sensor cells (GS-293/ EPAC-293). The proof of concept for such a system was established by initially using co-cultures of sensor cells with donor cell lines: KK-1 and FSHR-293, which express LHCGR and FSHR, respectively. The stimulation of LHCGR and FSHR by their respective ligands, LH and FSH, led to production of cAMP that was detected by the sensor cells (GS-293 or EPAC-203), leading to a luminescent/FRET read-out only in the presence of a phosphodiesterase inhibitor. Moreover, this method monitors cAMP accumulation over time as the assay medium contains IBMX and therefore cannot follow the desensitization process, since phosphodiesterases that are responsible for lowering the cAMP concentration in the cells are inhibited. Although 100 μM IBMX was used in CANDLES assay, higher concentrations of IBMX (200 μM) can also be used for low responding cells yet the background will also increase (Additional file 1: Figure S1). It was also established that the increase in luminescent signal from GS-293 cells is directly proportional to the number of donor cells and a 1:3 ratio of GS-293 cells to donor cells provides an optimal signal and can be used as a starting point for optimization of the sensor to donor cell ratios for other cell types. However, increasing the amount of sensor cells does not lead to increase in signal, rather it leads to a decrease in the overall luminescent signal, possibly as the number of cell-to-cell contact points between sensor and donor cells is reduced. Finally we tested the utility of this method in detecting cAMP production using two different primary cell cultures from rat cortical neurons and mouse granulosa cells. Moreover, fast kinetics of different agonists can easily be compared as was shown for isoproterenol, salbutamol and epinephrine stimulation of cortical neurons (note the duration of sharp peak in Figure 8C) that would otherwise be extremely difficult to compare by using competition-based assays.

For transferring of cAMP produced by the donor cells, cell-cell contact is essential as was demonstrated by the lack of luminescent signal generation upon physically separating the GS-293 from FSHR-293 cells, using permeable porous transwell chambers. The molecular components responsible for the transfer of cAMP were found to be gap junction channels. This was established from five different observations: first, the application of gap junction inhibitor, CBX, to co-cultures of GS-293 and FSHR-293 cells results in a decrease of the luminescent signal from GS-293 cells, after stimulation with rFSH, in a dose-dependent manner (Figure 6A). Second, this observed drop in luminescence could not be attributed to cytotoxicity, as incubation with CBX has no effect on cell viability. Third, when absolute cAMP concentrations in CBX-treated co-cultures of GS-293 and FSHR-293 cells were calculated after rFSH stimulation, it was found that cAMP production either remained similar (at lower CBX doses) or was increased (at higher CBX doses) in comparison to control co-cultures (without CBX treatment), thereby eliminating the possibility of a reduction in cAMP production. The apparent increase in cAMP following CBX treatment might be due to blockage of cAMP transfer to the neighboring cells and the cell culture medium, which usually happens in physiological conditions thereby causing the cell lysates to retain higher cAMP concentration. Equivalently, the entire kinetics of cAMP production upon CBX treatment followed in GS-293-FSHR cells (expressing both the receptor and the cAMP sensor) shows no significant changes in cAMP production after CBX addition. Thus the application of gap junction inhibitor in co-cultures of sensor and donor cells did not lead to a decrease in cAMP production but rather caused a blockage of transfer of cAMP from donor to sensor cells, thereby demonstrating that gap junction channels are responsible for transfer of cAMP in CANDLES assay. Finally, the overexpression of gap junction protein (Cx32) leads to an increase in cAMP detection by the sensor cells, thereby suggesting that gap junction channels are responsible for transfer of cAMP in CANDLES assay.

One of the major advantages of this method is the ability to kinetically monitor cAMP production, especially in primary cell cultures that are otherwise difficult to transfect and analyze with fluorescent or luminescent cAMP sensors available at present [19,22,28,29,31]. We therefore decided to generate separate biosensor cell lines expressing either a luminescent cAMP sensor (GloSensor 22F) or a FRET-based cAMP sensor (^T^EPAC^VV^). The luminescent sensor was chosen over FRET-based sensors to avoid issues relating to photobleaching, the requirement of a more complex plate-reader (and/or microscopes) and the need for extensive normalization controls. Moreover, the dose-response of a FRET based cAMP sensor (^T^EPAC^VV^) generated very similar changes in FRET ratio, making it difficult to compare the cAMP production in FSHR-293 cells stimulated with different doses of rFSH (Additional file 2: Figure S2). The application of a separate sensor cell line also solves the problem of high variability because of the use of different cell samples that are needed for multiple time-point measurements using competition based cAMP assays that require cell lysis. As this assay can measure cAMP production from endogenous receptors expressed in primary cell cultures, the response upon GPCR stimulation can be considered to be physiologically more relevant. Moreover, a dose-dependent response can be measured, which shows the specificity of the assay. CANDLES can also be used as a quick screening method to ascertain the expression of functional Gα_S_-coupling GPCRs in cell types that have been in cell culture for a prolonged duration, or to verify the identity and cross-contamination of cells with a different cell type, as recently reported in many laboratories [45].

One of the limitations of the assay is that the cells have to be equilibrated with the GloSensor cAMP reagent prior to stimulation of cells. This only allows multiple stimulations (with different drugs) of the cells to be in the same assay medium. However, this issue is common to all luminescence-based sensors, not CANDLES in particular. In addition, this assay cannot determine the absolute concentration of cAMP in the samples, and immunoassays still remain the method of choice if the aim is to determine absolute cAMP concentrations with high sensitivity. Nevertheless, EC_50_ values calculated using CANDLES assay are similar to those using ELISA. However, there is a possibility that some cell types may not form cell-cell contacts with sensor cells. Hence the applicability of different donor cells for the use with CANDLES has to be empirically determined.

## Conclusion

CANDLES obviate the limitations imposed by current cAMP assays that either cannot measure kinetics or requires inefficient cell transfections with cAMP sensors. The CANDLES assay is an easy and quick method to indirectly follow the entire cAMP generation curves from a variety of cell types including untransfected primary cells as well as from different receptors (LHCGR, FSHR, adrenergic receptors). Since assay measurements at room temperature (25-28°C) give the best signal-to-noise ratio (Additional file 4: Figure S4), only a single luminescent plate-reader is required, and sophisticated humidified gas chambers for live cell measurements are unnecessary. The assay is highly robust and measurements can easily be read for up to 2 hours. In addition, sensor cells can be adapted to grow in different cell culture media (DMEM/F12, RPMI-1640, McCoy’s 5A) ensuring proper growth conditions for further co-incubation with the primary cells of choice. Finally, the assay works for cells of different origins, for example primary cultured neurons from rats, granulosa cells from mice as well as human cells. The major advantage of this assay lies in experiments where monitoring the kinetics of cAMP production upon GPCR activation or following different cell stimuli is needed.

## Methods

### Cell culture

Human embryonic kidney-293 (HEK-293) cells were obtained from ATCC and KK-1 cell line (mouse granulosa cell line) was developed in our laboratory [41]. Cell lines were cultured in Dulbecco’s Modified Eagle Medium (DMEM)/F12 media (Gibco) supplemented with 10% fetal calf serum (FCS; PromoCell), 50 IU/ml penicillin and 50 μg/ml streptomycin (hereafter referred to as DMEM complete medium) and grown at 37**°**C in humidified atmosphere with 5% CO_2_. Cells (FSHR-293 and GS-293; see below) were adapted from DMEM complete medium to grow in RPMI 1640 or McCoy’s 5A media (Gibco) supplemented with 10% FCS, 50 IU/ml penicillin and 50 μg/ml streptomycin.

### Assay medium for luminescence measurements

Prior to experiments, cell culture medium was replaced with a freshly prepared assay medium. The assay medium was formulated with CO_2_-independent medium (Invitrogen), DMEM/F12, GloSensor cAMP reagent (Promega), 3-Isobutyl-1-methylxanthine (IBMX; Sigma) and Bovine serum albumin (BSA; Gibco). The assay medium contained a 1:1 ratio of DMEM/F12 (0.1% BSA) and CO_2_-independent media (0.1% BSA) supplemented with 2% GloSensor cAMP reagent and 100 μM IBMX.

### Generation of the stable luminescent cAMP-sensor (GS-293) cell line

HEK-293 cells in 6-well plates (3 × 10^5^ cells) were transfected with pGloSensor-22F cAMP plasmid (Promega) [31] using JetPEI transfection reagent (Polyplus transfection). The cells carrying the pGloSensor-22F plasmid were selected with hygromycin (200 μg/ml) in DMEM complete media. Following hygromycin selection, the selected clones were seeded in 96-well plates and grown until confluence, followed by testing for maximal luminescence after addition of 10 μM forskolin (LC laboratories, USA) in assay media without IBMX. This was repeated few times to select clones with highest response to cAMP generation. The best clones were then selected for three criteria: 1) highest luminescence upon addition of 10 μM forskolin; 2) low and consistent basal luminescence; and 3) consistent signal with serial passages. The final clone after four rounds of selection was subsequently named as HEK293-GloSensor (GS-293) and was used for all further experiments.

### Assay methodology with GS-293 sensor cells

GS-293 cells were co-cultured with other cells expressing the receptor under study (primary cell cultures or established cell lines) in either 12- or 24-well plates in DMEM complete medium for 24-48 h. Prior to experiment, DMEM complete medium was replaced with freshly prepared assay medium. Cells were equilibrated for 45 min at room temperature in dark. The cells were then kept at a constant temperature of 25**°**C in a plate reader (Victor, Perkin-Elmer-Wallac) for 20 min and luminescence was measured for 5 min to obtain a basal measurement. Measurements were made at 25**°**C because GloSensor-22F protein responds better at room temperature and the signal drastically decreases at 37**°**C (Additional file 4: Figure S4). Luminescence from individual wells was read either for 2, 5 or 10 s depending upon the intensity of signal. The assay was then performed by addition of specific ligands and recording luminescence values for up to 2 h (see Figure 2 for a quick protocol).

### Generation of stable FRET-based cAMP sensor cell line

A FRET-based cAMP sensor (^T^EPAC^VV^) was kindly provided by Prof. Kees Jalink [22]. HEK-293 cells were transfected with ^T^EPAC^VV^ sensor using JetPEI transfection reagent followed by selection in G418 antibiotic (400 μg/ml) containing medium. Cells were subsequently sorted for high expression of the sensor using BD FACSAria III cell sorter at Cell Imaging Core, Turku Centre for Biotechnology. After two rounds of cell sorting, the resultant sensor cell line was named as EPAC-293.

### Assay methodology with EPAC-293 cells and FRET analysis

EPAC-293 and FSHR-293 cells (50,000 cells each) were co-cultured in 24-well plates in DMEM complete medium. Prior to the assay, DMEM complete medium was replaced with DMEM/F12 medium without phenol red, supplemented with 100 μM IBMX and 0.1% BSA. Cells were incubated at room temperature for 15 min and subsequently transferred to Synergy H1 plate reader (BioTek) kept at room temperature (25**°**C). Cells were excited at 430/18 nm and fluorescence emission was read at 480/18 nm and 528/18 nm. FRET ratio was calculated by dividing fluorescent intensity values at 480 nm with intensity values at 528 nm. FRET values at the start of the experiment were set to 1.

### Isolation and culturing of cortical neurons

Primary cortical neurons were isolated from newborn Sprague-Dawley rats as previously described [46]. Briefly, dissociated neurons were plated in Minimal Essential Medium (MEM; Invitrogen) with 10% bovine calf serum (HyClone), 33 mM D-glucose, 2 mM L-glutamine, 50 IU/ml penicillin and 50 μg/ml streptomycin at the density 700,000 cells/cm^2^ on poly-D-Lysine (50 μM) (Sigma) coated 24-well plates (Greiner). Proliferation of non-neuronal cells was prevented by addition of 2.5 μM cytosine β-D-arabinofuranoside (Sigma) from the second day onward. Cells were maintained at 37**°**C with 5% CO_2_ until usage between 2 and 4 days in vitro. Neurons were stimulated with epinephrine, salbutamol, isoproterenol or glutamate (Sigma).

### Isolation and culturing of granulosa cells

Ovaries from 25-day-old C57BL/6 female mice were processed for follicular puncture as described previously [47] with some modifications. Briefly, ovaries were collected into DMEM/F12 medium without phenol red containing 50 IU/ml penicillin and 50 μg/ml streptomycin followed by incubation into DMEM/F12 medium containing 10 mM EGTA (Sigma) and 0.5M Sucrose for 30 min. Fresh DMEM/F12 medium was used to wash and collect ovaries. Granulosa cells were collected by puncturing the ovaries with a 25G needle 100 times in small volume of media under a dissecting microscope. Cells were passed through a 100 μm nylon cell strainer (BD Biosciences) to remove cell aggregates. Cells were centrifuged at 100 *X g* for 10 min and then resuspended in DMEM/F12 medium supplemented with 1 X insulin, transferrin, selenium solution (ITS-G; Gibco), 10% fetal bovine serum-charcoal stripped (Sigma), 50 IU/ml penicillin and 50 μg/ml streptomycin. 100,000 cells were seeded in a 12-well plate. Granulosa cells were stimulated with recombinant human luteinizing hormone (rLH; Organon).

### Gap junction analysis and cell viability

Carbenoxolone disodium salt (CBX; Sigma) was used as a gap junction inhibitor. Human connexin-32 plasmid (Cx32), C-terminally tagged with mEmerald, was obtained from Addgene (plasmid #54054) [48] while pcDNA 3.1 mock plasmid was purchased from Invitrogen. EVOS fluorescent microscope was used to verify the expression of Cx32 in cells (Additional file 3: Figure S3). Co-cultures of GS-293 and FSHR-293 cells were transiently transfected with Cx32 plasmid to determine the effect of connexin overexpression on cAMP transfer.

For determining cell viability after CBX treatment, co-cultures of GS-293 and FSHR-293 were preincubated with different concentrations of CBX (25, 50, 75 and 100 μM) for 2h in assay medium. Assay medium was then discarded, followed by washing with PBS. Cell viability was then assessed by CellTiter AQueous non-radioactive cell proliferation assay (Promega) following manufacturer’s instructions. Briefly, cells were incubated in DMEM complete medium (without phenol red) containing MTS/PMS [3-(4,5-dimethylthiazol-2-yl)-5-(3-carboxymethoxyphenyl)-2-(4-sulfophenyl)-2H-tetrazolium/phenazine methosulfate] solution for 2h (37°C, 5% CO_2_). Absorbance was then read at 490 nm, which provides a measure of cellular viability. One-way analysis of variance (ANOVA) was used to determine statistical differences among different samples.

### Cyclic AMP ELISA

A commercial cAMP ELISA kit (Cell Biolabs) was used to determine total cAMP levels following manufacturer’s instructions. Briefly, co-cultures of GS-293 and FSHR-293 (150,000 cells each) were seeded in 6-well plates. Prior to ELISA, cells were incubated in assay medium (without cAMP GloSensor reagent) containing different doses of CBX (25, 50, 75 and 100 μM) for 1h at room temperature. Cells were then stimulated with rFSH (200 mIU/ml) for 20 min and subsequently lysed. Cell lysates were used to calculate cAMP values (see Figure 1A for principle of cAMP ELISA kit). Total protein from cell lysates was calculated using Bicinchoninic Acid (BCA) protein assay kit (Thermo Scientific). cAMP concentrations were normalized to total cellular protein.

For calculating EC_50_ values, FSHR-293 cells (150,000 cells) were seeded in 6-well plates. Cells were incubated in DMEM/F12 (0.1% BSA, 100 µM IBMX) for 1h followed by stimulation with different doses of rFSH (0.1 mIU/ml to 1000 mIU/ml) for 20 min and then lysed. Cell lysate was similarly used to calculate cAMP concentrations. Total cAMP values were normalized to total cellular protein.

### Statistical analysis

Significance among samples was compared by one-way ANOVA. For EC_50_ calculation, cAMP values were normalized to percent maximal responses and curves were fitted using four-parameter logistic curve using PRISM 6 software.

### Ethical approval

All procedures were carried out according to the institutional and ethical policies of the University of Turku and approved by the local ethics committee on animal experimentation.

## Additional files

Additional file 1: Figure S1. Determination of IBMX concentration for CANDLES. Co-cultures of GS-293 and FSHR-293 (50,000 cells each) were preincubated in assay medium with different concentrations of IBMX (1, 10, 100 and 200 μM) for 45 min at room temperature and then transferred to plate reader kept at 25**°**C. Luminescence was then measured and cells were stimulated with rFSH (200 mIU/ml). Although 200 μM IBMX gives the highest signal upon rFSH stimulation of cells, it also has a higher background in unstimulated co-culture (red line). 100 μM IBMX also gives a high luminescent signal upon rFSH stimulation of cells but it has lower basal luminescence in unstimulated co-culture (green line) and was chosen as IBMX concentration of choice to be used in CANDLES. Data represented as mean of triplicates for one representative experiment (± SEM; negative direction) with at least three independent repeats. Additional file 2: Figure S2. FSH dose-response in co-culture of EPAC-293 with FSHR-293 cells. Co-culture of EPAC-293 and FSHR-293 (50,000 cells each) were stimulated with different doses of rFSH (10, 100, 200 and 1000 mIU/ml). Forskolin (10 μM) stimulation was used as a positive control while unstimulated co-cultures of EPAC-293 and FSHR-293 were used as negative control. Cells were excited at 430/18 nm and changes in FRET ratio (480/528 nm) was monitored by recording the fluorescence intensity at 480/18 and 528/18 nm. FRET ratio was set to 1 at the beginning of experiment. Increasing doses of rFSH (100, 200 and 1000 mIU/ml) yielded very similar changes in FRET ratio. Data represented as mean of triplicates for one representative experiment (± SEM; positive direction) with at least three independent repeats. Additional file 3: Figure S3. Cx32 expression in co-cultures of GS-293 and FSHR-293 cells. The expression of Connexin 32 tagged with mEmerald (Cx32-mEmerald) was verified in co-cultures of GS-293 and FSHR-293 cells. Merged phase contrast images and fluorescent images (mEmerald) are shown. pcDNA 3.1 was used as a mock control. Additional file 4: Figure S4. CANDLES assay responses at room temperature (25**°**C) and at 37°C. Co-cultures of GS-293 (50,000 cells) and FSHR-293 (50,000 cells) were incubated in assay medium either at 25**°**C or 37**°**C for 45 min and then transferred to plate reader kept at the same temperature for 20 min. Luminescence was then read and cells were stimulated with rFSH (200 mIU/ml). The sensor protein (GloSensor 22F) responds better at 25**°**C and both the signal as well as baseline drops significantly at 37°C (also stated in Promega’s manual [49]). Data represented as mean of triplicates for one representative experiment (± SEM) with at least three independent repeats.

## Abbreviations

CANDLES: *C*yclic *A*MP i*N*direct *D*etection by *L*ight *E*mission from *S*ensor cells
GPCRs: G protein-coupled receptors
cAMP: 3′,5′-cyclic adenosine monophosphate
ATP: Adenosine triphosphate
PKA: Protein kinase A
EPAC: Exchange protein activated by cAMP
FRET: Fluorescence resonance energy transfer
BRET: Bioluminescence resonance energy transfer
BSL-2: Biosafety level 2
LHCGR: Luteinizing hormone/chorionic gonadotropin receptor
HEK-293: Human embryonic kidney-293
FSHR: Follicle-stimulating hormone receptor
FSHR-293: HEK-293 cells stably transfected with FSHR
GS-293: HEK293-GloSensor
EPAC-293: HEK-293 cells stably transfected with ^T^EPAC^VV^ sensor
IBMX: 3-isobutyl-1-methylxanthine
rFSH: Recombinant human follicle-stimulating hormone
rLH: Recombinant human luteinizing hormone
DMEM: Dulbecco’s modified eagle medium
CBX: Carbenoxolone
ELISA: Enzyme-linked immunosorbent assay
GS-293-FSHR: GS-293 cells stably transfected with human FSHR
Cx32: Connexin 32
ANOVA: Analysis of variance
RLU: Relative Light Unit
RPMI: Roswell Park Memorial Institute
GS-293-LHCGR: GS-293 cells stably transfected with the LHCGR
FCS: Fetal calf serum
BSA: Bovine serum albumin
MEM: Minimal Essential Medium
MTS/PMS: 3-(4,5-dimethylthiazol-2-yl)-5-(3-carboxymethoxyphenyl)-2-(4-sulfophenyl)-2H-tetrazolium/phenazine methosulfate
BCA: Bicinchoninic Acid

## References

[1] Lagerstrom MC, Schioth HB (2008). Structural diversity of G protein-coupled receptors and significance for drug discovery. Nat Rev Drug Discov.

[2] Fredriksson R, Lagerstrom MC, Lundin LG, Schioth HB (2003). The G-protein-coupled receptors in the human genome form five main families. Phylogenetic analysis, paralogon groups, and fingerprints. Mol Pharmacol.

[3] Sato M, Blumer JB, Simon V, Lanier SM (2006). Accessory proteins for G proteins: partners in signaling. Annu Rev Pharmacol Toxicol.

[4] Wettschureck N, Offermanns S (2005). Mammalian G proteins and their cell type specific functions. Physiol Rev.

[5] Tilley DG (2011). G protein-dependent and G protein-independent signaling pathways and their impact on cardiac function. Circ Res.

[6] Gsandtner I, Charalambous C, Stefan E, Ogris E, Freissmuth M, Zezula J (2005). Heterotrimeric G protein-independent signaling of a G protein-coupled receptor. Direct binding of ARNO/cytohesin-2 to the carboxyl terminus of the A2A adenosine receptor is necessary for sustained activation of the ERK/MAP kinase pathway. J Biol Chem.

[7] Sunahara RK, Dessauer CW, Gilman AG (1996). Complexity and diversity of mammalian adenylyl cyclases. Annu Rev Pharmacol Toxicol.

[8] Chung KY, Rasmussen SG, Liu T, Li S, DeVree BT, Chae PS, Calinski D, Kobilka BK, Woods VL, Sunahara RK (2011). Conformational changes in the G protein Gs induced by the beta2 adrenergic receptor. Nature.

[9] Rasmussen SG, DeVree BT, Zou Y, Kruse AC, Chung KY, Kobilka TS, Thian FS, Chae PS, Pardon E, Calinski D, Mathiesen JM, Shah ST, Lyons JA, Caffrey M, Gellman SH, Steyaert J, Skiniotis G, Weis WI, Sunahara RK, Kobilka BK (2011). Crystal structure of the beta2 adrenergic receptor-Gs protein complex. Nature.

[10] Katritch V, Cherezov V, Stevens RC (2013). Structure-function of the G protein-coupled receptor superfamily. Annu Rev Pharmacol Toxicol.

[11] Nichols WW (1970). Virus-induced chromosome abnormalities. Annu Rev Microbiol.

[12] Reddel RR, Ke Y, Gerwin BI, McMenamin MG, Lechner JF, Su RT, Brash DE, Park JB, Rhim JS, Harris CC (1988). Transformation of human bronchial epithelial cells by infection with SV40 or adenovirus-12 SV40 hybrid virus, or transfection via strontium phosphate coprecipitation with a plasmid containing SV40 early region genes. Cancer Res.

[13] Jin Y, Mertens F, Mandahl N, Heim S, Olegard C, Wennerberg J, Biorklund A, Mitelman F (1993). Chromosome abnormalities in eighty-three head and neck squamous cell carcinomas: influence of culture conditions on karyotypic pattern. Cancer Res.

[14] Degorce F, Card A, Soh S, Trinquet E, Knapik GP, Xie B (2009). HTRF: A technology tailored for drug discovery - a review of theoretical aspects and recent applications. Curr Chem Genomics.

[15] Bradley J, McLoughlin D (2009). Use of the DiscoveRx Hit hunter cAMPII assay for direct measurement of cAMP in Gs and Gi GPCRs. Methods Mol Biol.

[16] Gabriel D, Vernier M, Pfeifer MJ, Dasen B, Tenaillon L, Bouhelal R (2003). High throughput screening technologies for direct cyclic AMP measurement. Assay Drug Dev Technol.

[17] Brooker G, Terasaki WL, Price MG (1976). Gammaflow: a completely automated radioimmunoassay system. Science.

[18] Steiner AL, Kipnis DM, Utiger R, Parker C (1969). Radioimmunoassay for the measurement of adenosine 3′,5′-cyclic phosphate. Proc Natl Acad Sci U S A.

[19] Willoughby D, Cooper DM (2008). Live-cell imaging of cAMP dynamics. Nat Methods.

[20] Taylor SS, Yang J, Wu J, Haste NM, Radzio-Andzelm E, Anand G (2004). PKA: a portrait of protein kinase dynamics. Biochim Biophys Acta.

[21] Gloerich M, Bos JL (2010). Epac: defining a new mechanism for cAMP action. Annu Rev Pharmacol Toxicol.

[22] Klarenbeek JB, Goedhart J, Hink MA, Gadella TW, Jalink K (2011). A mTurquoise-based cAMP sensor for both FLIM and ratiometric read-out has improved dynamic range. PLoS One.

[23] Mongillo M, McSorley T, Evellin S, Sood A, Lissandron V, Terrin A, Huston E, Hannawacker A, Lohse MJ, Pozzan T, Houslay MD, Zaccolo M (2004). Fluorescence resonance energy transfer-based analysis of cAMP dynamics in live neonatal rat cardiac myocytes reveals distinct functions of compartmentalized phosphodiesterases. Circ Res.

[24] Ponsioen B, Zhao J, Riedl J, Zwartkruis F, van der Krogt G, Zaccolo M, Moolenaar WH, Bos JL, Jalink K (2004). Detecting cAMP-induced Epac activation by fluorescence resonance energy transfer: Epac as a novel cAMP indicator. EMBO Rep.

[25] Zaccolo M, Pozzan T (2002). Discrete microdomains with high concentration of cAMP in stimulated rat neonatal cardiac myocytes. Science.

[26] Nikolaev VO, Bunemann M, Hein L, Hannawacker A, Lohse MJ (2004). Novel single chain cAMP sensors for receptor-induced signal propagation. J Biol Chem.

[27] Zhang J, Ma Y, Taylor SS, Tsien RY (2001). Genetically encoded reporters of protein kinase A activity reveal impact of substrate tethering. Proc Natl Acad Sci U S A.

[28] Zaccolo M, De Giorgi F, Cho CY, Feng L, Knapp T, Negulescu PA, Taylor SS, Tsien RY, Pozzan T (2000). A genetically encoded, fluorescent indicator for cyclic AMP in living cells. Nat Cell Biol.

[29] Jiang LI, Collins J, Davis R, Lin KM, DeCamp D, Roach T, Hsueh R, Rebres RA, Ross EM, Taussig R, Fraser I, Sternweis PC (2007). Use of a cAMP BRET sensor to characterize a novel regulation of cAMP by the sphingosine 1-phosphate/G13 pathway. J Biol Chem.

[30] Prinz A, Diskar M, Erlbruch A, Herberg FW (2006). Novel, isotype-specific sensors for protein kinase A subunit interaction based on bioluminescence resonance energy transfer (BRET). Cell Signal.

[31] Binkowski BF, Butler BL, Stecha PF, Eggers CT, Otto P, Zimmerman K, Vidugiris G, Wood MG, Encell LP, Fan F, Wood KV (2011). A luminescent biosensor with increased dynamic range for intracellular cAMP. ACS Chem Biol.

[32] Warrier S, Belevych AE, Ruse M, Eckert RL, Zaccolo M, Pozzan T, Harvey RD (2005). Beta-adrenergic- and muscarinic receptor-induced changes in cAMP activity in adult cardiac myocytes detected with FRET-based biosensor. Am J Physiol Cell Physiol.

[33] Sosinsky GE, Nicholson BJ (2005). Structural organization of gap junction channels. Biochim Biophys Acta.

[34] Herve JC, Derangeon M, Sarrouilhe D, Giepmans BN, Bourmeyster N (1818). Gap junctional channels are parts of multiprotein complexes. Biochim Biophys Acta.

[35] Giepmans BN (2004). Gap junctions and connexin-interacting proteins. Cardiovasc Res.

[36] Bedner P, Niessen H, Odermatt B, Willecke K, Harz H (2003). A method to determine the relative cAMP permeability of connexin channels. Exp Cell Res.

[37] Lawrence TS, Beers WH, Gilula NB (1978). Transmission of hormonal stimulation by cell-to-cell communication. Nature.

[38] Kam Y, Kim DY, Koo SK, Joe CO (1998). Transfer of second messengers through gap junction connexin 43 channels reconstituted in liposomes. Biochim Biophys Acta.

[39] Bevans CG, Kordel M, Rhee SK, Harris AL (1998). Isoform composition of connexin channels determines selectivity among second messengers and uncharged molecules. J Biol Chem.

[40] Ponsioen B, van Zeijl L, Moolenaar WH, Jalink K (2007). Direct measurement of cyclic AMP diffusion and signaling through connexin43 gap junctional channels. Exp Cell Res.

[41] Kananen K, Markkula M, Rainio E, Su JG, Hsueh AJ, Huhtaniemi IT (1995). Gonadal tumorigenesis in transgenic mice bearing the mouse inhibin alpha-subunit promoter/simian virus T-antigen fusion gene: characterization of ovarian tumors and establishment of gonadotropin-responsive granulosa cell lines. Mol Endocrinol.

[42] Liu J, Siragam V, Chen J, Fridman MD, Hamilton RM, Sun Y (2014). High-throughput measurement of gap junctional intercellular communication. Am J Physiol Heart Circ Physiol.

[43] Davidson JS, Baumgarten IM (1988). Glycyrrhetinic acid derivatives: a novel class of inhibitors of gap-junctional intercellular communication. Structure-activity relationships. J Pharmacol Exp Ther.

[44] Spray DC, Rozental R, Srinivas M (2002). Prospects for rational development of pharmacological gap junction channel blockers. Curr Drug Targets.

[45] Capes-Davis A, Theodosopoulos G, Atkin I, Drexler HG, Kohara A, MacLeod RA, Masters JR, Nakamura Y, Reid YA, Reddel RR, Freshney RI (2010). Check your cultures! A list of cross-contaminated or misidentified cell lines. Int J Cancer.

[46] Bjorkblom B, Ostman N, Hongisto V, Komarovski V, Filen JJ, Nyman TA, Kallunki T, Courtney MJ, Coffey ET (2005). Constitutively active cytoplasmic c-Jun N-terminal kinase 1 is a dominant regulator of dendritic architecture: role of microtubule-associated protein 2 as an effector. J Neurosci.

[47] Burkart AD, Mukherjee A, Mayo KE (2006). Mechanism of repression of the inhibin alpha-subunit gene by inducible 3′,5′-cyclic adenosine monophosphate early repressor. Mol Endocrinol.

[48] Fort AG, Murray JW, Dandachi N, Davidson MW, Dermietzel R, Wolkoff AW, Spray DC (2011). In vitro motility of liver connexin vesicles along microtubules utilizes kinesin motors. J Biol Chem.

[49] **GloSensor™ cAMP Assay Technical Manual** [https://www.promega.com/~/media/files/resources/protocols/technical%20manuals/0/glosensor%20camp%20assay%20protocol.pdf]

