# Supplementary material for: CANDLES, an assay for monitoring GPCR induced cAMP generation in cell cultures
Source: Cell Commun Signal. 2014 Nov 4;12:70. doi: 10.1186/s12964-014-0070-x (PMC4228090; doi:10.1186/s12964-014-0070-x)
Supplement: Additional file 1: Figure S1. — Determination of IBMX concentration for CANDLES. Co-cultures of GS-293 and FSHR-293 (50,000 cells each) were preincubated in assay medium with different concentrations of IBMX (1, 10, 100 and 200 μM) for 45 min at room temperature and then transferred to plate reader kept at 25°C. Luminescence was then measured and cells were stimulated with rFSH (200 mIU/ml). Although 200 μM IBMX gives the highest signal upon rFSH stimulation of cells, it also has a higher background in unstimulated co-culture (red line). 100 μM IBMX also gives a high luminescent signal upon rFSH stimulation of cells but it has lower basal luminescence in unstimulated co-culture (green line) and was chosen as IBMX concentration of choice to be used in CANDLES. Data represented as mean of triplicates for one representative experiment (± SEM; negative direction) with at least three independent repeats. [file 12964_2014_70_MOESM1_ESM.pdf]

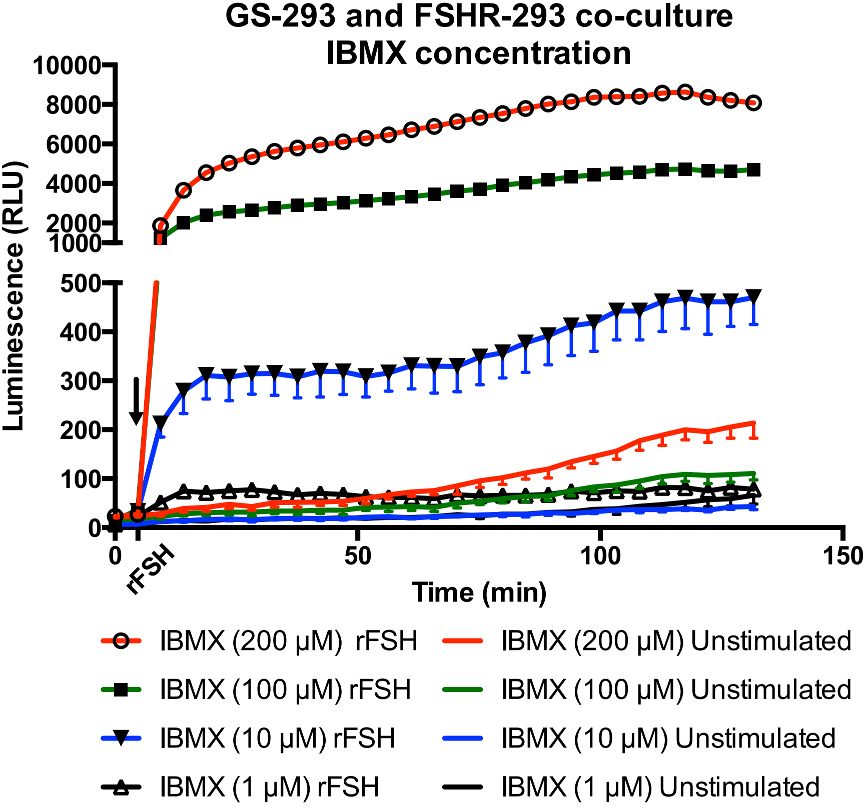
GS-293 and FSHR-293 co-culture
IBMX concentration
Luminescence (RLU)
10000
8000
6000
4000
2000
1000
500
400
300
200
100
0
0
rFSH
50
100
150
Time (min)
IBMX (200 μM) rFSH
IBMX (200 μM) Unstimulated
IBMX (100 μM) rFSH
IBMX (100 μM) Unstimulated
IBMX (10 μM) rFSH
IBMX (10 μM) Unstimulated
IBMX (1 μM) rFSH
IBMX (1 μM) Unstimulated
