# Supplementary material for: CANDLES, an assay for monitoring GPCR induced cAMP generation in cell cultures
Source: Cell Commun Signal. 2014 Nov 4;12:70. doi: 10.1186/s12964-014-0070-x (PMC4228090; doi:10.1186/s12964-014-0070-x)
Supplement: Additional file 2: Figure S2. — FSH dose-response in co-culture of EPAC-293 with FSHR-293 cells. Co-culture of EPAC-293 and FSHR-293 (50,000 cells each) were stimulated with different doses of rFSH (10, 100, 200 and 1000 mIU/ml). Forskolin (10 μM) stimulation was used as a positive control while unstimulated co-cultures of EPAC-293 and FSHR-293 were used as negative control. Cells were excited at 430/18 nm and changes in FRET ratio (480/528 nm) was monitored by recording the fluorescence intensity at 480/18 and 528/18 nm. FRET ratio was set to 1 at the beginning of experiment. Increasing doses of rFSH (100, 200 and 1000 mIU/ml) yielded very similar changes in FRET ratio. Data represented as mean of triplicates for one representative experiment (± SEM; positive direction) with at least three independent repeats. [file 12964_2014_70_MOESM2_ESM.pdf]

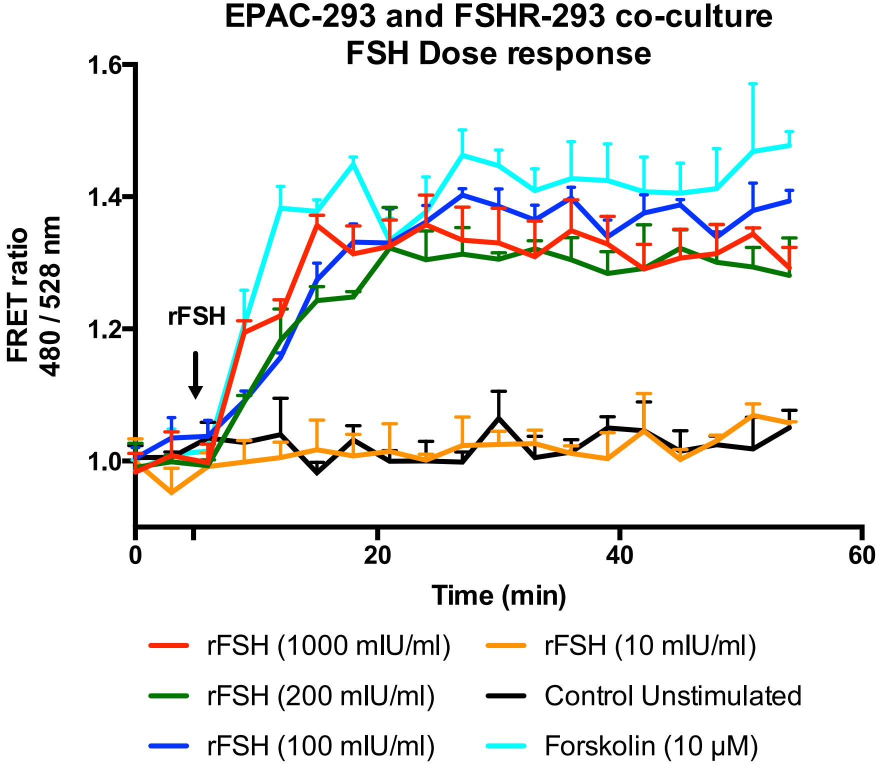

EPAC-293 and FSHR-293 co-culture
FSH Dose response
FRET ratio
480 / 528 nm
1.6
1.4
1.2
1.0
rFSH
0
20
40
60
Time (min)
rFSH (1000 mIU/ml)
rFSH (200 mIU/ml)
rFSH (100 mIU/ml)
rFSH (10 mIU/ml)
Control Unstimulated
Forskolin (10 µM)
