# Supplementary figures and images for: CANDLES, an assay for monitoring GPCR induced cAMP generation in cell cultures
Source: Cell Commun Signal. 2014 Nov 4;12:70. doi: 10.1186/s12964-014-0070-x (PMC4228090; doi:10.1186/s12964-014-0070-x)

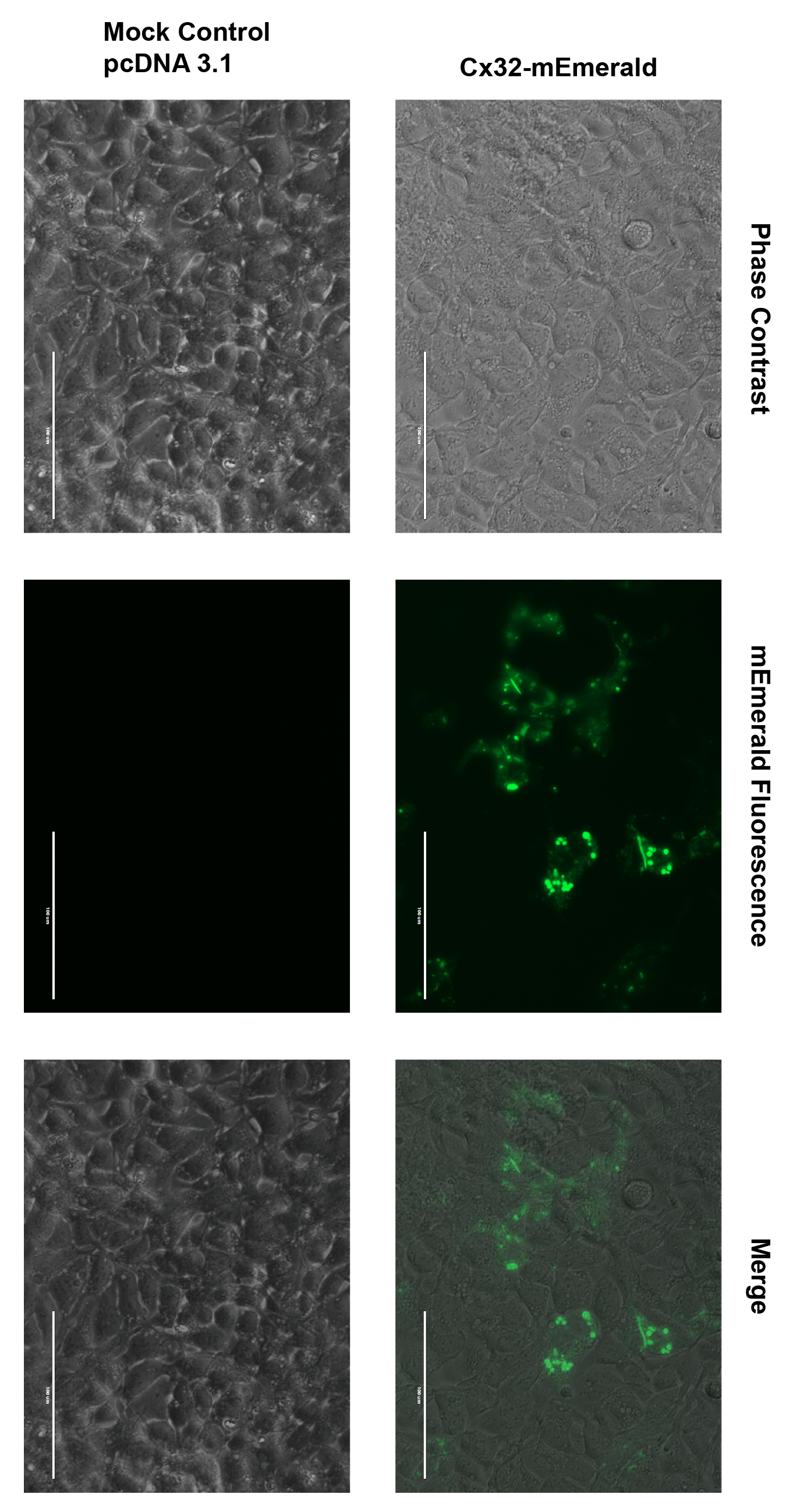

Supplement: Additional file 3: Figure S3. — Cx32 expression in co-cultures of GS-293 and FSHR-293 cells. The expression of Connexin 32 tagged with mEmerald (Cx32-mEmerald) was verified in co-cultures of GS-293 and FSHR-293 cells. Merged phase contrast images and fluorescent images (mEmerald) are shown. pcDNA 3.1 was used as a mock control. [file 12964_2014_70_MOESM3_ESM.tiff]
