# Supplementary material for: CANDLES, an assay for monitoring GPCR induced cAMP generation in cell cultures
Source: Cell Commun Signal. 2014 Nov 4;12:70. doi: 10.1186/s12964-014-0070-x (PMC4228090; doi:10.1186/s12964-014-0070-x)
Supplement: Additional file 4: Figure S4. — CANDLES assay responses at room temperature (25°C) and at 37°C. Co-cultures of GS-293 (50,000 cells) and FSHR-293 (50,000 cells) were incubated in assay medium either at 25°C or 37°C for 45 min and then transferred to plate reader kept at the same temperature for 20 min. Luminescence was then read and cells were stimulated with rFSH (200 mIU/ml). The sensor protein (GloSensor 22F) responds better at 25°C and both the signal as well as baseline drops significantly at 37°C (also stated in Promega’s manual [49]). Data represented as mean of triplicates for one representative experiment (± SEM) with at least three independent repeats. [file 12964_2014_70_MOESM4_ESM.pdf]

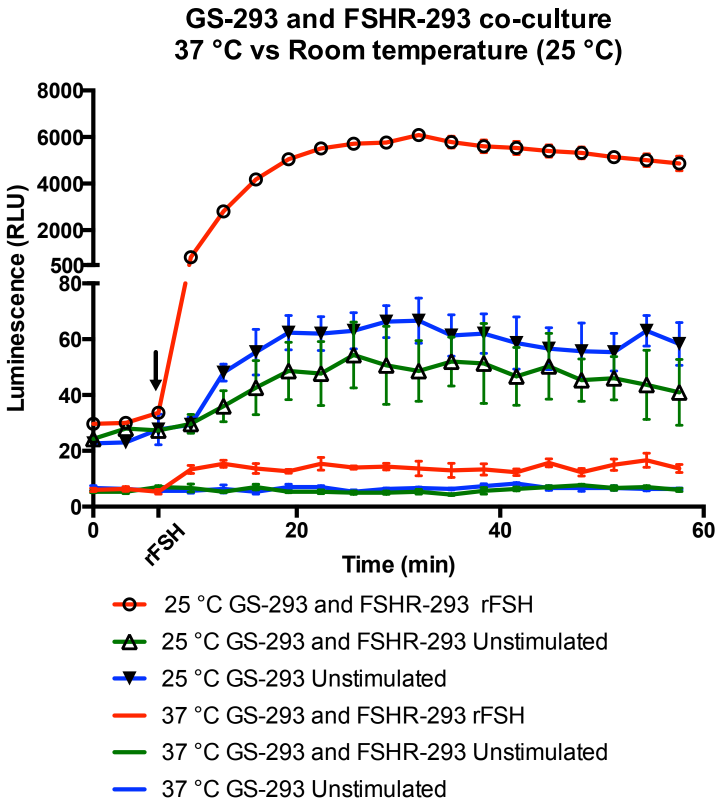
GS-293 and FSHR-293 co-culture
37 °C vs Room temperature (25 °C)
Luminescence (RLU)
8000
6000
4000
2000
500
80
60
40
20
0
0
rFSH
20
40
60
Time (min)
25 °C GS-293 and FSHR-293 rFSH
25 °C GS-293 and FSHR-293 Unstimulated
25 °C GS-293 Unstimulated
37 °C GS-293 and FSHR-293 rFSH
37 °C GS-293 and FSHR-293 Unstimulated
37 °C GS-293 Unstimulated
